# Epicutaneous Exposure to Staphylococcal Superantigen Enterotoxin B Enhances Allergic Lung Inflammation via an IL-17A Dependent Mechanism

**DOI:** 10.1371/journal.pone.0039032

**Published:** 2012-07-27

**Authors:** Jinho Yu, Min Hee Oh, Ju-Un Park, Allen C. Myers, Chen Dong, Zhou Zhu, Tao Zheng

**Affiliations:** 1 Division of Allergy and Clinical Immunology, Johns Hopkins University School of Medicine, Baltimore, Maryland, United States of America; 2 Department of Immunology, Center for Inflammation and Cancer, MD Anderson Cancer Center, Houston, Texas, United States of America; Louisiana State University, United States of America

## Abstract

Atopic dermatitis (AD) is the initial step of the atopic march: the progression from AD to allergic rhinitis and asthma. There is a close association between skin barrier abnormalities and the development of AD and the atopic march. One of cardinal features of AD is that the lesional skin of the majority of AD patients is chronically colonized with *Staphylococcus aureus* with half isolates producing superantigen enterotoxin B (SEB). Although diverse roles of SEB in the pathogenesis and severity of AD have been recognized, whether SEB contributes to the dermal inflammation that drives lung inflammation and airway hyperresponsiveness (AHR) has not been examined. Here we show a novel role of *S. aureus* superantigen SEB in augmenting allergen ovalbumin (Ova) induced atopic march through an IL-17A dependent mechanism. When mice epicutaneously (EC) sensitized with allergen Ova, addition of topical SEB led to not only augmented systemic Th2 responses but also a markedly exaggerated systemic Th17/IL-17 immune environment. The ability of SEB in enhancing Th17/IL-17 was mediated through stimulating lymphocytes in spleen and draining lymph nodes to promote IL-6 production. Epicutaneous sensitization of mice with a combination of Ova and SEB significantly enhanced Ova-induced AHR and granulocytic lung inflammation than Ova allergen alone. When IL-17A was deleted genetically, the effects of SEB on augmenting lung inflammation and AHR were markedly diminished. These findings suggest that chronic heavy colonization of enterotoxin producing *S. aureus* in the skin of patients with atopic dermatitis may have an important role in the development of atopic march via an IL-17A dependent mechanism.

## Introduction

The hallmarks of atopic dermatitis (AD), also termed atopic eczema, include chronic, pruritic, relapsing form of skin inflammation, disturbance of epidermal-barrier function that culminates in dry skin, and IgE-mediated sensitization to environmental allergens [1]. AD is considered an entry point of the atopic march, the progression of atopic disorder from AD in infants to allergic rhinitis and finally to asthma in children and adults and underlying atopy is considered the thread linking these disorders [2], [3], [4], [5], [6], [7]. The concept of a progressive atopic march is supported by multiple lines of genetic, epidemiological and experimental evidence. These studies indicate that reduced filaggrin expression in the human skin leading to impaired epidermal barrier function can be a major predisposing factor for AD and subsequent development of the atopic march [8], [9], [10]. Epidemiologic data from cross sectional and longitudinal studies support the sequential development from AD to asthma [11], [12], [13], [14], [15]. The progression from AD to asthma was also supported by experimental data showing that allergen exposure through the epidermis can initiate systemic allergy [16]. These data suggest a causal link between childhood eczema and the later-onset respiratory allergic disorders. However, the underlying mechanisms of the atopic march are largely unknown.

One cardinal feature of AD is remarkable susceptibility to colonization with *Staphylococcus aureus* (*S. aureus*). The skin of 90% of patients with AD is colonized with *S. aureus* as compared with only 10% of healthy individuals [17], [18]. Approximately 50% of isolated *S. aureus* produce superantigens including enterotoxin B (SEB) [19], [20], [21]. The severity of dermatitis correlates with both the number of colonized *S. aureus* [22] and the presence of superantigen-producing *S. aureus* [21], [23]. The capacity of superantigens to cause massive stimulation of T cells and macrophages, Langerhans cells, and activated keratinocytes accounts for most of their pathologic effects [24]. In addition to directly activating T cells via T cell receptor (TCR) Vβ chains in an allergen-independent manner, superantigens can augment T-helper 1 (Th1) response in an antigen-specific manner via the induction of interleukin-12 (IL-12) in antigen-presenting cells (APCs), which might contribute to AD while becoming chronic [24], [25]. SEB-activated dendritic cells (DC) drive Th2 cell differentiation by activating DCs via Toll-like receptor 2 (TLR-2) [26]. In addition, the SEB superantigen is shown to enhance house dust mite-induced patch test allergic reactions in patients with AD [27], [28]. Mouse models of AD using repeated epicutaneous sensitization with ovalbumin (Ova) to tape-stripped skin has been reported [16], [29]. It has been shown that topical SEB superantigen exposure in the skin induces mixed Th1/Th2 type dermatitis and production of IgE antibodies in a murine model of atopic dermatitis in wild type mice on BALB/c genetic background [30]. Although the diverse roles of SEB in the pathogenesis and severity of AD have been well appreciated, its role in the atopic march is unknown.

Th17 cells produce IL-17A (or IL-17), IL-17F and IL-22 [27]. Th17 cells and IL-17A have recently been implicated in the pathogenesis of AD and asthma. Humans who lack Th17 cells are more susceptible to *S. aureus* infections compared to individuals who have Th17 cells [31]. IL-17A induces the expression of neutrophil-attracting chemokines, such as CXCL2 [32], and recruitment of neutrophils [33]. Epicutaneous (EC) immunization of mice with Ova allergen promotes IL-17 expression in the skin and drives the generation of IL-17 producing T cells in the inguinal, axillary and cervical lymph nodes (DLNs) and spleen and a local and systemic Th17 response associated with neutrophilic airway inflammation [34], [35]. Moreover, Infiltrating T cells isolated from the atopy patch test reactions from AD patients can produce IL-17 and SEB superantigen strongly promoted IL-17 release by T cells in culture [36]. It has been demonstrated that IL-17A present in the lung, either produced by transgenic over-expression or induced by allergens, is able to induce lung inflammation, mucous metaplasia and airway hyperresponsiveness [37], [38].

In the present study, we showed that epicutaneous immunization of mice with a combination of Ova and SEB exhibited significantly enhanced skin inflammation, particularly IL-17A than Ova-sensitization alone, suggesting that SEB contributes to Ova-induced AD. We further investigated the effect of SEB superantigen on pulmonary inflammation and airway hyperresponsiveness in mice that were epicutaneously immunized and airway challenged with Ova allergen. In addition, we examined the role of IL-17A in the atopic march in mice epicutaneously sensitized with a combination of Ova and SEB.

## Results

### SEB caused increased dermal IL-17A and enhanced Ova-induced skin inflammation

**Figure 1:**
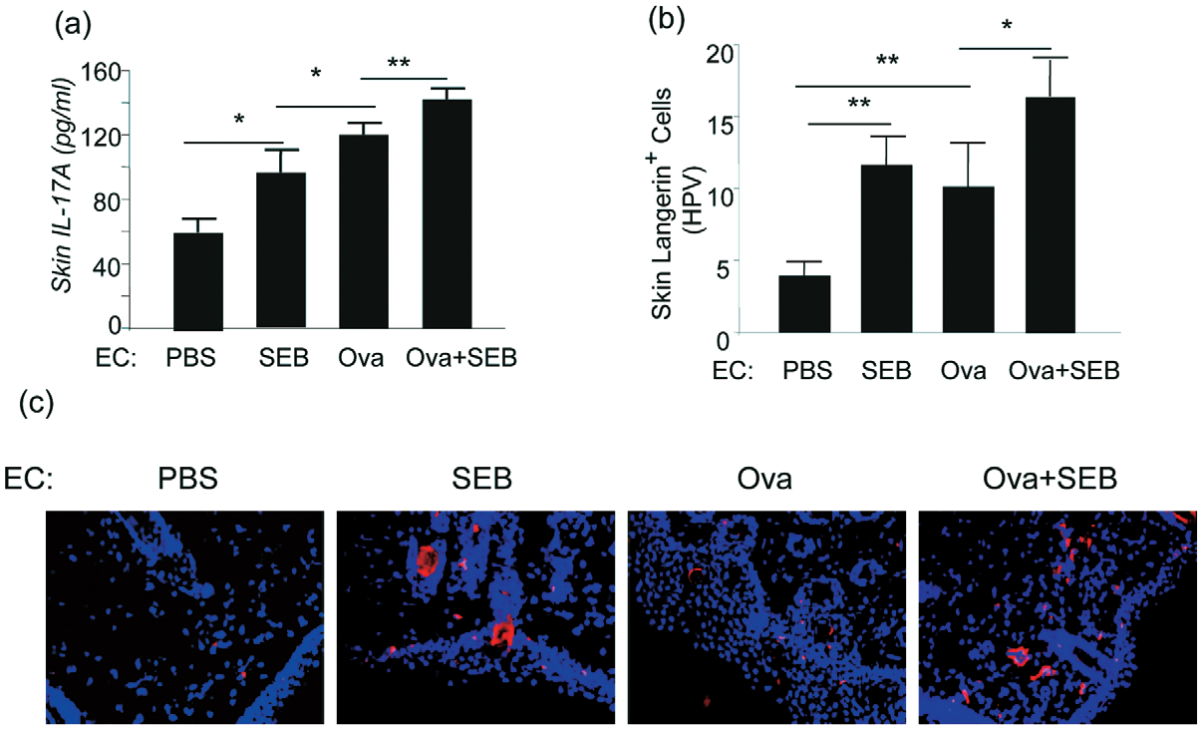
SEB enhanced IL-17A production and Langerhans cell accumulation in the skin. Mice were epicutaneously sensitized with PBS, SEB, Ova, or Ova + SEB and airway challenged with Ova. Skin samples were prepared for proteins and histology. (a) IL-17A was measured by ELISA of the skin protein extracts (n = 7–8 for each group; *p<0.05, **p<0.001). (b) Immunofluorescence (IF) staining of Langerin-positive Langerhans cells in the skin and quantification under high power view (n = 5 for each group; *p≤0.05, **p≤0.001). (c) IF of Langerhans cells in the skin (representatives of at least 5 samples for each group).

Mouse models of AD using repeated epicutaneous sensitization with Ova to tape-stripped skin have been reported [16], [29]. SEB superantigen exposure in the skin induces mixed Th1/Th2 type dermatitis and production of IgE antibodies in a murine model of atopic dermatitis in wild type mice on BALB/c background [30]. Using the same protocol of EC-sensitization but in mice on C57BL/6 background (**Figure S1a**), we found that the skin of wild type mice sensitized with Ova + SEB exhibited increased epidermal thickness with hyperplasia and increased inflammatory responses compared with PBS, Ova, or SEB sensitized groups (**Figure S1b. c. d**). In line with other studies, in the skin of PBS sensitized mice a few inflammatory cells were seen scattered in the dermis, whereas in Ova or SEB sensitized skin there was significantly increased dermal inflammation (**Figure S1b**). However, further increased granulocytes, including eosinophils and neutrophils were seen in the dermis of Ova + SEB sensitized mice (**Figure S1e**). Examination of cytokine profile by ELISA in sensitized skin samples showed that EC-SEB sensitization and EC-Ova sensitization induced production of IL-4, IL-13 but not IFN-γ (**Figure S1f-h**). In addition, EC sensitization of wild type mice with either SEB or Ova alone caused increased dermal IL-17A production and the levels of IL-17A were significantly augmented in mice sensitized with Ova + SEB (**Figure 1a**). On the other hand, IL-17F, another member of the IL-17 cytokine family, was not detected in the skin of these mice. Furthermore, by immunofluorescence (IF), SEB sensitized skin showed increased Langerin positive Langerhans cells in the dermal regions compared to PBS or Ova treated skin. There was a further increase in Langerhans cells in the dermis of Ova + SEB treated skin (**Figure 1b, 1c**), suggesting that SEB strongly activates or mobilize Langerhans cells, which are normally located in the epidermis and may be able to migrate through dermis towards the lymphatic vessels to the draining lymph nodes.

### SEB augmented epicutaneous Ova-induced lung inflammation and AHR

**Figure 2:**
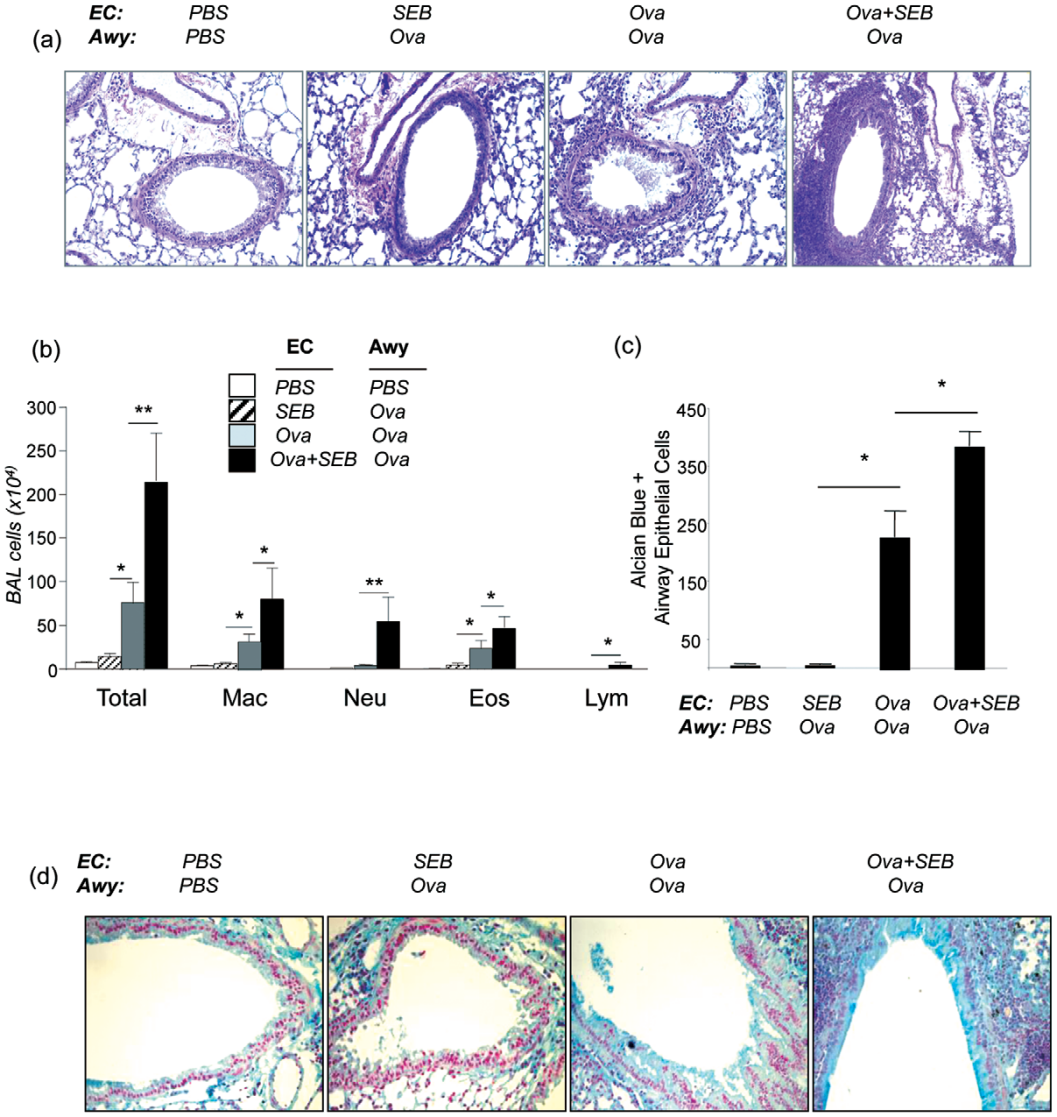
SEB enhanced pulmonary inflammation after Ova airway challenge. Mice were epicutaneously sensitized with PBS, SEB, Ova, Ova + SEB and challenged with Ova as described in Methods. BAL and lung samples were obtained for analysis. (a) H&E staining of lung sections examined at magnification ×20 and ×200. (b) Total and differential cell counts in the bronchoalveolar lavage fluid (BAL) (n = 5–7 per group; *p≤0.05, **p≤0.01). (c) Quantification of Alcian blue positive airway epithelial cells (n = 4–5 for each group; *p≤0.05). (d) Alcian blue stained lung sections of PBS, SEB, Ova, Ova + SEB EC-sensitized and airway Ova challenged mice (representative slides for 5 samples per group).

Previous studies showed that airway challenge of epicutaneous Ova sensitized wild type (BALB/c) mice caused airway Th2 inflammation and AHR [34], [35]. We investigated the potential effects of epicutaneous exposure of SEB on Ova induced lung inflammation, airway mucus metaplasia and AHR following Ova inhalation challenge in wild type (C57BL/6) mice. After intranasal challenge (Airway or Awy) with Ova per protocol shown in **Figure S1a**, there were minimal inflammatory cells (mononuclear cells) seen in the lung tissues (H&E) of EC-SEB sensitized mice (EC-SEB/Awy-Ova) and EC-PBS sensitized mice (EC-PBS/Awy-PBS). However, lungs from EC-Ova + SEB sensitized mice (EC-Ova + SEB/Awy-Ova) exhibited markedly increased inflammatory cells in peribronchial and perivascular areas compared to EC-Ova sensitized mice (EC-Ova/Awy-Ova) (**Figure 2a**)**.** Consistently, the numbers of total cells and macrophages, lymphocytes and in particular, neutrophils in the BAL samples from EC-Ova + SEB/Awy-Ova mice were significantly increased compared to those corresponding cell types in EC-Ova/Awy-Ova mice. There were also more eosinophils in the Ova-SEB group than those in the Ova group, although the difference did not reach statistical significance. The total and differential cell counts in EC-SEB/Awy-Ova and EC-PBS/Awy-PBS mice were similar at baseline (**Figure 2b**).

Mucous hyperplasia as another hallmark of asthma was assessed by Alcian blue staining. In the airways of EC-PBS/Awy-PBS and EC-SEB/Awy-Ova mice Alcian blue positive cells were rarely seen, whereas positive cells were readily seen in the airway of EC-Ova/Awy-Ova mice. However, EC-Ova + SEB/Awy-Ova mice exhibited further increased Alcian blue positive cells in the airways, indicating enhanced mucus hyperplasia in these mice (**Figure 2c and 2d**). These data showed that epicutaneous sensitization with SEB together with Ova significantly enhances lung inflammation characterized by eosinophil rich and neutrophilic predominant inflammation and airway mucous hyperplasia.

**Figure 3:**
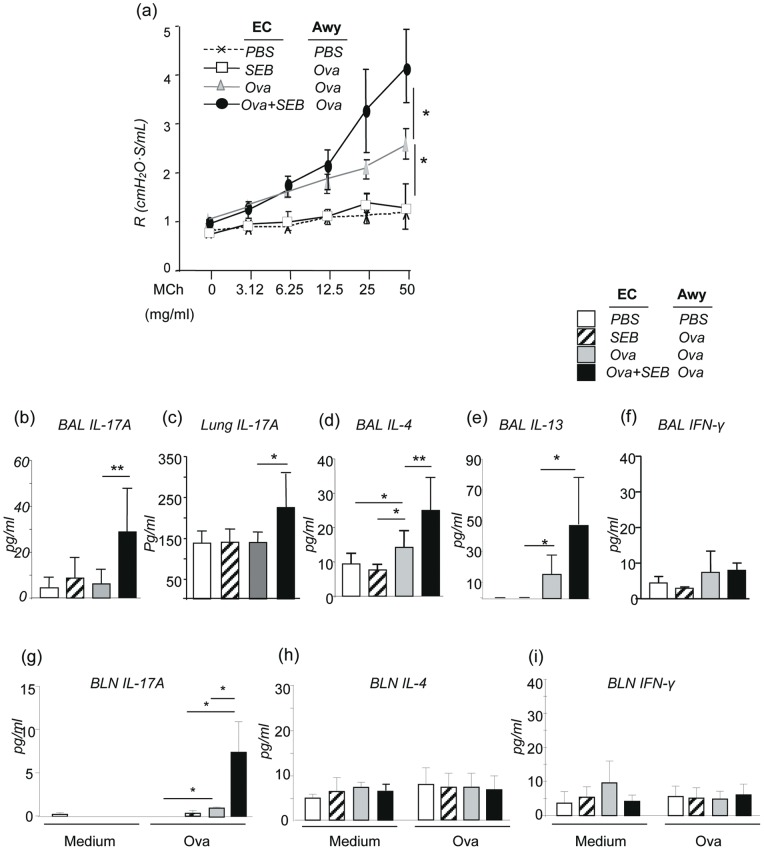
SEB enhanced airway resistance and Th17 and Th2 cytokine expression in the lung of EC-sensitized and Ova airway challenged mice. Airway resistance (*R_L_*) in response to increasing concentrations of methacholine was measured by the invasive PFT method (flexiVent). Cytokines in the BAL and lung tissues were measured by ELISA. (a) Changes in airway resistance (shown are combined data from two separate experiments; n = 4–5 per group; *p<0.05). (b, d, e, f) IL-17A, IL-4, IL-13, and IFN-γ in the BAL; (c) IL-17A in lung tissue; and (g, h, i) Lymphocytes of bronchial lymph nodes produced IL-17A, IL-4, and IFN-γ, respectively (n = 7 each group; *p≤0.05, **p≤0.01).

Using an invasive PFT method we determined the lung resistance (R_L_) of sensitized and challenged mice [39]. The PBS control mice and EC-SEB sensitized and Ova airway challenged mice showed no or minimum responses to methacholine challenge. EC-Ova/Awy-Ova mice displayed increased AHR compared to the control groups. However, EC-Ova + SEB/Awy-Ova mice showed further exaggerated AHR to methacholine stimulation compared to EC-Ova/Awy-Ova mice (**Figure 3a**).

As expected, Ova sensitized and challenged EC-Ova/Awy-Ova mice showed increased Th2 cytokines IL-4 and IL-13 but not Th1 cytokine IFN-γ or Th17 cytokine IL-17A in the BAL, whereas EC-PBS/Awy-PBS and EC-SEB/Awy-Ova mice showed only basal levels of these cytokines (**Figure 3b, d-i**). When SEB was combined with Ova during epicutaneous sensitization, there were further increases in the levels of IL-4, IL-13, but not IFN-γ in the BAL. Furthermore, IL-17A was increased in the BAL of these mice (**Figure 3b, d–i**). The increase in IL-17A was also seen in the lung tissue of EC-Ova + SEB sensitized mice, although there were moderate basal levels of IL-17A in all groups (**Figure 3c**). The amount of IL-17A produced by lymphocytes from bronchial draining lymph nodes of EC-Ova + SEB/Awy-Ova mice was significantly higher than that in EC-Ova/Awy-Ova or EC-SEB/Awy-Ova mice (**Figure 3g**). The amounts of IL-4 and IFN-γ produced by lymphocytes were not different among all groups (**Figure 3h-i**). The amount of IL-6 in the BAL was not significantly increased in EC-SEB/Awy-Ova and EC-Ova/Awy-Ova mice but IL-6 was significantly more in the BAL of EC-Ova + SEB/Awy-Ova mice than the other 3 groups (**Figure 3b**).

When viewed together, epicutaneous sensitization of wild type mice with superantigen SEB enhanced epicutaneous Ova-induced lung inflammation, airway mucus metaplasia, AHR and Th2 and Th17 cytokine production upon airway Ova allergen challenge.

### SEB stimulated and enhanced IL-17A production by lymphocytes from EC-SEB and EC- Ova + SEB sensitized mice

**Figure 4:**
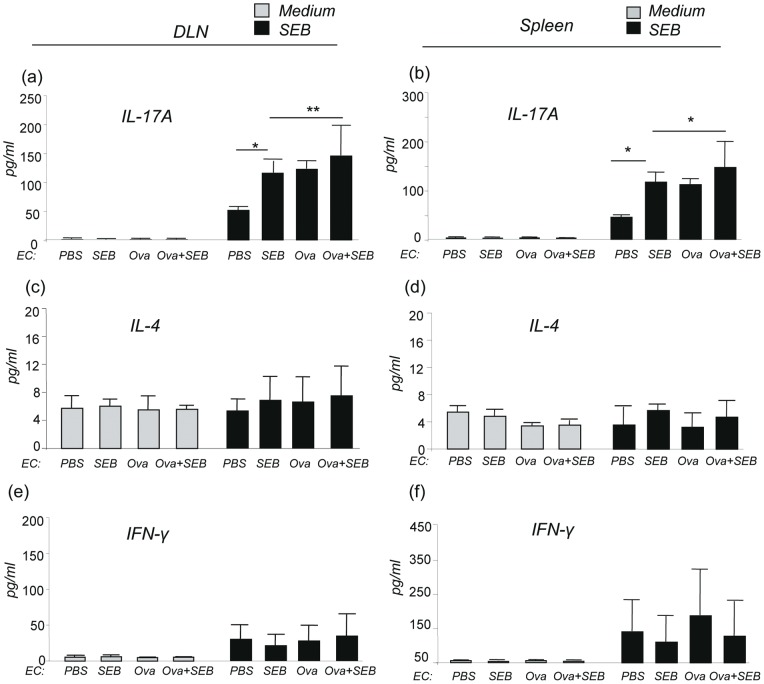
SEB stimulated lymphocyte IL-17A production. IL-17A, IL-4 and IFN-γ produced by lymphocytes from cervical, axillary, inguinal draining lymph nodes (DLNs) or from spleen after SEB stimulation (a-f). Data are representative of two experiments with similar results. Columns and error bars represent Mean ± SEM (n = 5–7 per group; *p≤0.05, **p≤0.01).

**Figure 5:**
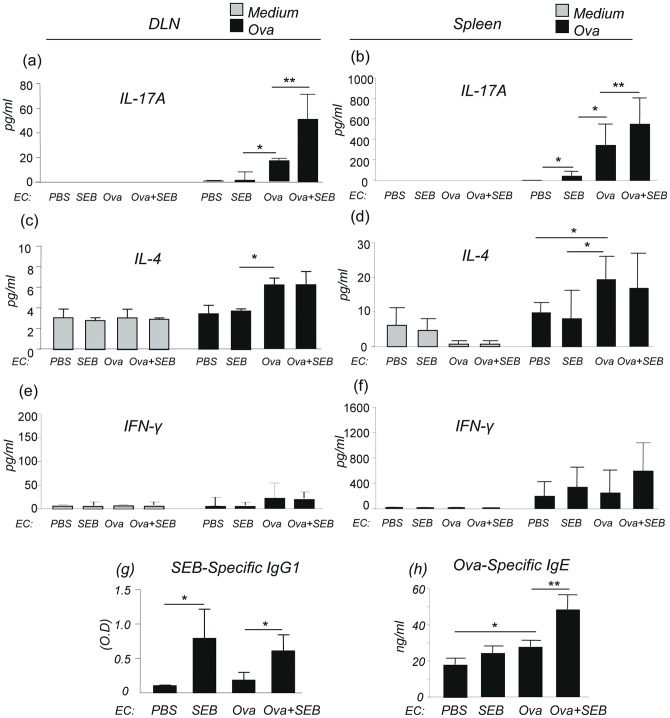
SEB synergistically enhanced Ova stimulated IL-17A production by lymphocytes and exaggerated systemic Ova-specific IgE production. (a–f) Ova stimulated production of IL-17A, IL-4, and IFN-γ by lymphocytes from DLN or spleen of mice sensitized with PBS, SEB, Ova and Ova + SEB (n = 5–7 per group; *p≤0.05, **p≤0.01). (g) SEB-specific IgG1 and (h) Ova-specific IgE in the serum. Data are Mean ± SEM (n = 5–7 per group; *p≤0.05, **p≤0.01).

It has been shown that epicutaneous immunization of mice with Ova triggers activation of IL-17-producing T cells in the draining lymph nodes and spleen [34]. We investigated whether epicutaneous SEB could directly stimulate lymphocytes to produce IL-17 and enhance Ova induced systemic Th17 responses. In medium control samples only background levels of cytokines were detected (**Figure 4a-f**). After stimulation with SEB (50 ng/ml) for 72 hrs, lymphocytes from the DLNs and spleen of EC-SEB sensitized and EC-Ova sensitized mice produced significantly higher amounts of IL-17A, but not IL-4 or IFN-γ, as compared to those from EC-PBS mice (**Figure 4a, 4c and 4e**), indicating that SEB directly activates lymphocytes of the DLNs and spleen to produce IL-17A. A further increase in the IL-17A levels was seen in EC-Ova + SEB sensitized mice (**Figure 4a, 4b**). Lymphocytes from the DLNs and spleen of EC-Ova-sensitized mice stimulated with Ova for 72 hrs produced more IL-17A than those from EC-SEB and EC-PBS sensitized groups (**Figure 5a, 5b**), and importantly, there was a synergistic increase in IL-17A by lymphocytes of DLN and spleen from EC-Ova + SEB sensitized mice (**Figure 5a and 5b**). Ova stimulated lymphocytes of the DLNs and spleen from EC-Ova sensitized mice produced more IL-4 compared to EC-PBS mice and EC-SEB mice (**Figure 5c, 5d**). On the other hand, lymphocytes from all groups produced comparable levels of IFN-γ (**Figure 5e, 5f**)**.** However, IL-17A was undetectable in the serum samples of these mice (data not shown). These studies indicate that SEB directly stimulates lymphocytes of EC-PBS sensitized and EC-SEB sensitized mice to produce IL-17A, not IL-4 or IFN-γ, and SEB enhances production of IL-17A by lymphocytes from EC-Ova + SEB sensitized mice. These data also indicate that epicutaneous SEB exposure enhances EC-Ova sensitization-induced dermatitis probably by inducing cutaneous IL-17A and by acting additively and/or synergistically with Ova to promote exaggerated Th17/Th2 biased inflammatory responses.

We next evaluated the effects of SEB exposure on Ova sensitization-induced systemic immune responses. Similar to EC-SEB sensitized mice, EC-Ova + SEB sensitized mice showed highly increased levels of SEB-specific IgG1 compared to EC-Ova and EC-PBS sensitized groups (**Figure 5g**)**.** While the levels of Ova-specific IgE in EC-Ova mice were higher than those of EC-PBS and EC-SEB sensitized mice, SEB exposure significantly increased the levels of Ova-specific IgE in EC-Ova + SEB sensitized mice (**Figure 5h**).

### SEB enhanced EC-Ova-induced Th17 immunity by augmenting IL-6 production from lymph nodes and spleen

**Figure 6:**
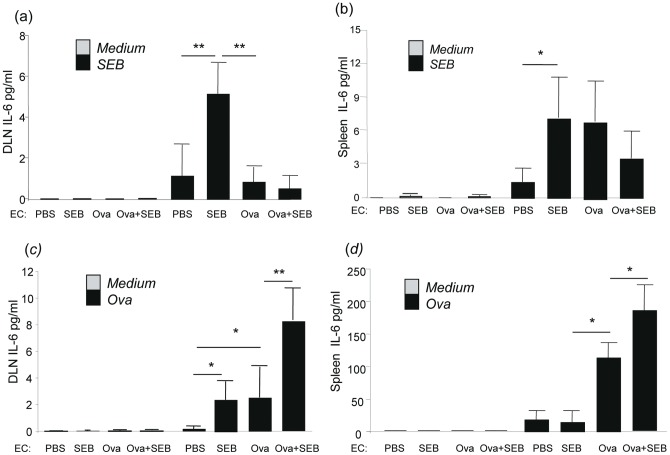
SEB stimulated and enhanced Ova induced IL-6 production by immune cells. (a, b) SEB stimulated IL-6 production by lymphocytes from skin DLNs and spleen for 72 hrs. (c, d) Ova stimulated IL-6 produced by lymphocytes from skin DLNs and spleen for 72 hrs. Data are representative of two experiments with similar results. Columns and error bars represent Mean ± SEM (n = 5–9 mice per group; *p≤0.05, **p≤0.01).

It is known that IL-6, transforming growth factor-β (TGF-β), and IL-23 are the major inducers of Th17 cells from naïve precursors [40], [41]. To further understand the mechanisms of how epicutaneous exposure of SEB stimulates Th17/IL-17A immunity, which led to enhanced AHR and neutrophil-predominant lung inflammation, we assessed the levels of IL-6, TGF-β1 and IL-23 secreted by lymphocytes of draining lymph nodes and spleen. After SEB stimulation for 72 hrs, DLN lymphocytes from EC-SEB sensitized mice produced markedly increased IL-6 compared to that by cells from EC-PBS, EC-Ova and EC-Ova + SEB sensitized mice (**Figure 6a**). Similarly, splenocytes stimulated with SEB for 72 hrs showed higher levels of IL-6 in EC-SEB and EC-Ova sensitized mice than those in EC-PBS sensitized mice, but comparable with those in EC-Ova + SEB sensitized mice (**Figure 6b**). After Ova stimulation for 72 hrs, lymphocytes of DLNs from EC-SEB, EC-Ova sensitized mice produced significantly more IL-6 than those from EC-PBS mice and there was a synergistic augmentation of IL-6 production by DLN lymphocytes from EC-Ova + SEB mice. Ova stimulated splenocytes displayed a similar pattern of IL-6 production, except that EC-SEB mice did not produce more IL-6 more EC-PBS mice (**Figure 6d**). On the other hand, the levels of TGF-β and IL-23 produced by lymphocytes stimulated by either SEB or Ova were not significantly altered among all groups (**Figure S2**). These data suggest that superantigen SEB stimulates DLN lymphocytes and splenocytes to produce IL-6 and epicutaneous SEB exposure works independently as well as synergistically with Ova to augment IL-6, but not TGF-β or IL-23, production in response to Ova in an antigen specific fashion, favoring a polarized Th17/IL-17A immunity. The serum levels of IL-6 were similar at the baseline in all groups (**Figure S3a**).

### SEB induced augmentation of EC-Ova–induced lung inflammation and AHR requires IL-17A

**Figure 7:**
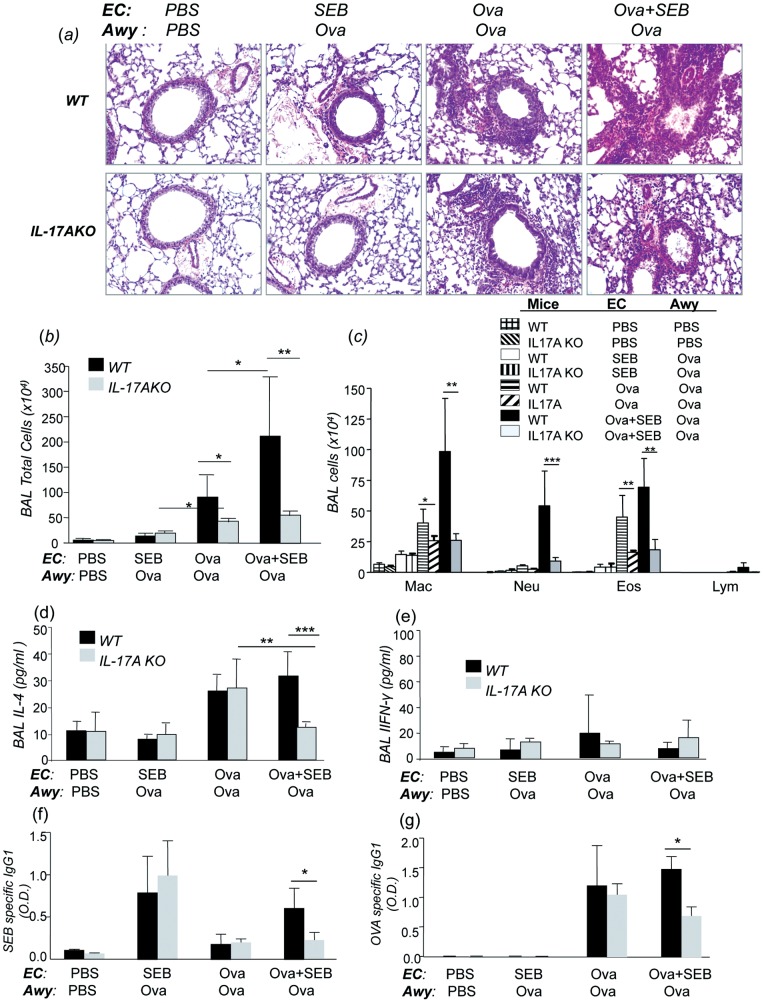
Effects of IL-17A gene disruption on SEB enhanced Ova-induced pulmonary inflammation, cytokine production and immunoglobulin production. Measurements were performed after EC-sensitization with PBS, SEB, Ova, or Ova + SEB and Ova airway challenge in IL-17A KO mice and compared with wild type mice. (a) H&E staining of lung sections examined at magnification ×20 (representatives of at least 5 samples each group). (b, c) Total and differential cell counts in the BAL and (d, e) IL-4 and IFN-γ levels in the BAL. (f, g) SEB-specific IgG1 and Ova-specific IgG1. Data shown are Mean ± SEM (n = 5 for each group; *p<0.05 and **p<0.01).

**Figure 8:**
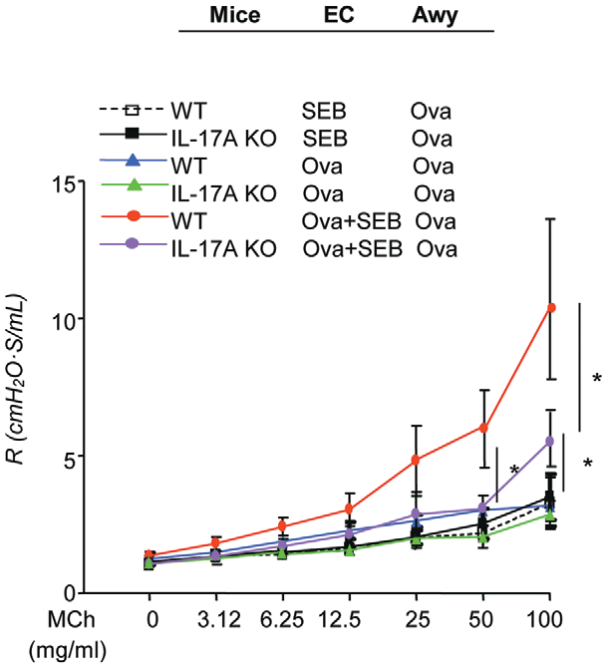
Deletion of IL-17A gene reduced SEB enhanced Ova-induced changes in lung physiology. (a) Airway resistance in response to methacholine after epicutaneous sensitization and Ova airway challenge. The columns and error bars represent Mean ± SEM (shown are combined data from two separate experiments; n = 4–5 per group; *p<0.05, compared to WT mice).

Our data above showed that epicutaneous sensitization with Ova in the presence of superantigen SEB drove the production of IL-17A in the skin, and by lymphocytes of draining lymph nodes and spleen, which was associated with exaggerated pulmonary inflammation characterized by mixed neutrophilic and eosinophilic inflammation and exaggerated AHR following Ova challenge. To further understand the involvement of IL-17A, we investigated the role of IL-17A in the SEB augmented Ova induced lung inflammation and AHR using IL-17A deficient mice (IL-17A KO) as compared to age-matched wild type C57BL/6 mice using the same experimental protocol. BAL and lung samples were collected 24 hrs after the last dose of Ova challenge, histological examination (H&E) of lungs from IL-17A KO mice epicutaneously sensitized with Ova + SEB revealed markedly reduced lung inflammation (**Figure 7a**), reduced total cells (**Figure 7b**) and differential cell counts in the BAL, particularly in neutrophilic and eosinophilic responses compared to wild type mice (**Figure 7c**). Markedly reduced BAL IL-4, serum SEB-specific IgG1 and Ova-specific IgE, but not INF-γ, were also found in EC-Ova + SEB sensitized mice lacking IL-17A (**Figure 7d-g**) compared to similarly sensitized and challenged wild type mice. EC-Ova sensitized mice lacking IL-17A also showed reduced level of lung inflammation and notably, eosinophils in the BAL (**Figure 7a, 7b, 7c**) compared to WT mice sensitized with Ova. EC-PBS and EC-SEB sensitized IL-17A KO mice exhibited similar levels of lung inflammation following Ova-challenge. Furthermore, deletion of the IL-17A gene significantly reduced systemic immunoglobulin production including serum SEB-specific IgG1 and Ova-specific IgG1 (**Figure 7f, 7g**)**.** Consistently, genetic deletion of IL-17A in mice that were epicutaneously sensitized with a combination of Ova and SEB greatly diminished AHR upon methacholine challenge compared to WT mice immunized with Ova + SEB, suggesting that IL-17A may be essential in triggering lung inflammation and AHR (**Figure 8**)**.** However, deletion of IL-17A in these mice did no alter airway mucus metaplasia (data nor shown). Together, these data indicate that epicutaneous allergen Ova immunization in the presence of superantigen SEB promotes neutrophil-predominant inflammatory responses in the lung following airway Ova challenge via an IL-17A-dependent mechanism.

## Discussion

Atopic dermatitis (AD), the most common skin disorder in children is considered the initial step of the atopic march: the progression from AD to allergic rhinitis and asthma. In human AD, bacterial products from *Staphylococcus aureus*, including superantigens and cell wall components are involved in the immune response in the pathogenesis of AD [42]. Extensive chronic colonization of superantigen-producing *S. aureus*, such as enterotoxin B (SEB), strongly correlates with the severity of AD [21], [23]. SEB is involved in the pathogenesis of AD in several ways. SEB is known not only to induce polyclonal T cell proliferation, but also to stimulate allergen-specific responses [43], probably through activation of dendritic cells via TLR-2 signaling to drive polarization of naïve T cells to Th2 cells [26]. SEB exacerbates AD by inhibiting the function of T regulatory cells that normally control inflammation [44]. Furthermore, a recent study showed that in a model of AD, topical exposure of SEB enhances Ova-induced mixed Th2/Th1 dermatitis [30].

The aim of this study was to investigate the role of epicutaneous exposure of superantigen SEB in Ova-induced atopic march. EC exposure of SEB stimulated a systemic Th17/IL-17 immune environment and enhanced EC-Ova induced systemic Th2 immune responses. This was partially through direct stimulation of increased lymphocyte production of IL-6, the inducer of Th17 differentiation from naïve T cells. Collectively, these changes led to eosinophil rich and neutrophil predominant lung inflammation and airway hyperresponsiveness. Furthermore, when the IL-17A gene was deleted, the contribution of SEB to EC-Ova induced atopic march was diminished. These data suggest that SEB plays an important role in Ova-induced lung inflammation and AHR via an IL-17A-dependent pathway.

Epicutaneous exposure of SEB in wild type mice induced cutaneous IL-17A and IL-4 and enhanced allergen Ova-induced IL-17A production, leading to Th2/Th17 dermal inflammation as well as Ova-specific IgE and SEB-specific IgG1 systemic responses. These findings suggest that SEB exacerbates or contributes to the persistent skin inflammation thereby enhancing allergen-specific immune responses as a superantigen and a pathogen-associated molecular pattern when coexisting with allergen. Increased expression of IL-17A was found in the acute skin lesions of patients with AD [45]. Topical sensitization of mice with Ova allergen together with superantigen SEB followed by airway allergen challenge led to significant neutrophil predominant and eosinophil rich lung inflammation and enhanced AHR that were associated with elevated IL-17A levels in the lung tissue, in the BAL and in the bronchial draining lymph nodes. Notably, enhanced systemic Th17 was associated with increased levels of IL-6, but not TGF-β and IL-23, produced by SEB stimulated or Ova stimulated lymphocytes from DLN and spleen of these mice (**Figure S2**), suggesting that IL-6 contributes to IL-17 immune responses. Increased IL-17A has been associated with neutrophilic airway inflammation in murine asthma models and in human studies [34], [46], [47], [48]. Transgenic expression of IL-17A in the airway induced lung inflammation, mucous metaplasia, and airway fibrosis in mice [37] and IL-17A may have direct effects on airway smooth muscle and may be responsible for allergen induced AHR [38]. The findings in our study indicate that epicutaneous sensitization with superantigen SEB not only plays an important role in the pathogenesis of AD but also in the development of AHR and lung inflammation following airway allergen challenge primarily by promoting Th17/IL-17. Isolates of S. aureus from patients with AD can produced different superantigens, including SEA, SEB, SEC, and TSST-1 [49], [50]. Whether the findings on SEB in this study have broad implications for other superantigens in the atopic march is not known and should be further investigated.

In a murine epicutaneous antigen (Ova) challenge model analogous to human AD, IL-17 expression was induced not only in the skin but also in the airways, which was independent of IL-4 and IL-13 [34], implicating IL-17 in the atopic march. Our studies showed that there was synergistic enhancement of IL-17A by epicutaneous co-sensitization of allergen Ova and superantigen SEB, suggesting that IL-17A may contribute to the atopic march. TGF-β, IL-6 and have been shown to be the major inducers of Th17 cells that produce IL-17 [40], [51], [52]. IL-6 is produced by various types of cells such as T cells, B cells, keratinocytes and endothelial cells [40]. IL-23, primarily produced by macrophages and DCs, is an important stimulator of Th17 differentiation, and IL-23R is found on memory T cells, NKT cells, macrophages, DCs, and naive T cells [53]. In the present study, markedly increased IL-6, not TGF-β and IL-23, produced by lymphocytes of spleen and draining lymph nodes in an allergen (Ova) specific manner (**Figure 5** **and Figure S2**) from mice epicutaneously sensitized with SEB and Ova was likely responsible for the enhanced Th17/IL-17 immunity that heightened the magnitude of the atopic march. Furthermore, the levels of IL-6 were significantly increased in the airways following Ova allergen sensitization and challenge in the EC-Ova + SEB mice, although no difference was noticed in the serum levels of IL-6 (**Figure S3**). Increased IL-6 in the lung may also contribute to the enhanced IL-17 response seen in the lung tissue of EC-Ova + SEB mice (**Figure 3b, c**).

In this study we further demonstrated that epicutaneous sensitization with SEB increases migration of epidermal Langerhans cells to the dermal area towards the draining lymph nodes and SEB directly stimulates lymphocytes of spleen and draining lymph nodes to produce IL-17A as well as synergistically works with allergen (Ova) in biasing to systemic Th17 immunity. When mice lacking IL-17A were epicutaneously co-sensitized with Ova and SEB, lung inflammation, particularly neutrophilic inflammation, to a less extent eosinophilic inflammation, and AHR were greatly attenuated, though not completely abolished, suggesting that topical SEB coexisting with allergen Ova can synergistically promotes Th17/IL-17 immunity that serves as a primary driver in causing lung inflammation and AHR. These results are consistent with findings by our group and others that IL-17A can induce airway inflammation and AHR in different model systems [37], [38].

Although the mechanism of the atopic march is largely unknown, EC sensitization to inhalant allergens in the individual with skin barrier defect followed by Th2/Th17-mediated systemic inflammation with subsequent airway inflammation has been suggested. As microbial co-exposure is required to develop allergic inflammation with allergen, superantigen SEB secreting *S. aureus* could be particularly important for the progression to the development of asthma in patients with AD whose skin is often colonized with these bacteria. To our knowledge, this study is the first to identify SEB from *S. aureus* having a role in the development of atopic march and involving IL-17 immunity. It remains to be further investigated whether targeting *S. aureus* colonization can halt the development of asthma in patients with atopic dermatitis.

## Materials and Methods

### Ethics Statement

All animal experiments were approved by the IACUC of the Johns Hopkins University.

### Animals and epicutaneous sensitization and airway challenge

C57BL/6 mice (6-8 weeks old, sex-matched) were purchased from the Jackson Laboratory (Bar Harbor, ME). IL-17A knockout mice (6–8 weeks old) were generated as described [54] and backcrossed to C57BL/6 background for more than 10 generations. Mice were kept under specific pathogen-free conditions. Using the protocol previously described [16] and in Figure S1a, 6–8 weeks-old mice were epicutaneously (EC) treated with PBS, Ova, SEB, or a combination of Ova and SEB. The back of the anesthetized mice was shaved with a razor and tape-stripped four times using adhesive tape to introduce skin injury. SEB (Toxin Technology, Inc, Sarasota, FL), 10 μg in 50 μL of PBS, Ova (grade V; Sigma, St. Louis, MO) 100 μg in 50 μL of PBS, or a combination of SEB and Ova in 50 μL of PBS was placed on a patch of sterile gauze (1×1 cm), which was then secured to the skin with a transparent bio-occlusive dressing. Each mouse received a total of three one-week exposures to the patch, separated with 2-week intervals.

### Measurement of cytokines and preparation of skin and lung tissue extracts

Protein samples were prepared as described previously [55]. Briefly, frozen skin and lung tissues were placed in liquid nitrogen, crushed with a mortar and pestle and weighed. Triton X-100 0.25% (wt/vol) in 1 ml PBS was added to 100 mg of the skin powder. For lung extracts, the whole lung from each mouse was treated with proteinase inhibitors in 1 ml of PBS containing Triton X-100 0.25% (wt/vol). The homogenate was stirred at 4°C overnight and then centrifuged at 3000×g for 15 minutes to remove debris. Supernatants were stored in small aliquots at −80°C until assayed. All samples were normalized to the weight of the skin samples. Cytokines in the skin and lung samples were determined using ELISA kits per the manufacturer's instructions (R&D Systems, Minneapolis, MN).

### Measurement of serum immunoglobulins

Serum samples for OVA-specific IgE, IgG1 and SEB-specific IgG1 were measured using ELISA Plates coated with OVA antigen (100 μg/ml, Sigma Aldrich), SEB (1 μg/ml, Toxin Technology, Inc) in 0.1 M carbonate-bicarbonate buffer (pH 9.5) overnight at 4°C. Plates were washed with PBS-Tween 20 (0.05%) and blocked with 3% BSA/PBS for 2 hrs at room temperature (RT). Plates were washed and diluted serum samples in 1% BSA/PBS were incubated 2 hrs at RT. After washing, biotin-conjugated rat-anti mouse IgE or IgG1 antibody (BD Bioscience) was incubated for 2 hrs at RT. Subsequently streptavidin-horseradish peroxidase was incubated for 30 min at RT. After washing, substrate solution (BD Bioscience) was added and the plates were read with ELISA reader.

### Airway challenge and assessment of pulmonary physiology

One day after the third epicutaneous sensitization (Protocol in Figure S1a), airway challenge was performed in WT and IL-17A deficient mice (IL-17AKO) by intranasal (i.n.) instillation of Ova (50 μg in 20 μL of PBS) once a day for 3 consecutive days. One day after the last dose of Ova challenge lung physiology was assessed using the invasive pulmonary function test as described previously [39]. The baseline total lung resistance and AHR to methacholine (MCh) challenge were assessed using invasive PFT for airway resistance (R) (cm H_2_O•s/ml) with a small rodent PFT apparatus flexiVent (SCIREQ Inc., Montreal, Canada) as descripted [39]. Mch (Sigma, St. Louis, MO) at various concentrations was delivered through an in-line nebulizer and dose-response curves to inhaled MCh were determined. After each dose, data were collected at 1-minute intervals and then averaged. The values for airway resistance were plotted as a function of MCh doses.

### Lung tissue and bronchoalveolar lavage fluid (BAL) samples

Lung tissue and BAL samples were obtained as previously described by our laboratories [56], [57]. Briefly, mice were anesthetized; the trachea was isolated by blunt dissection; and a small caliber tubing was inserted and secured in the airway. Three successive volumes of 0.7 ml of PBS were instilled and gently aspirated and pooled. BAL samples were centrifuged, and supernatants were stored at −70°C until assayed. Cells in 100 μL aliquots were counted. For cell differential, a total of 100,000 viable BAL cells were centrifuged onto slides using Cytospin 3 (Thermo Shandon Ltd, Runcorn, UK) and stained with Diff-Quik staining kit (Siemens Corporation, Washington, D.C.). The numbers and types of cells in the pellet were determined. The lung was perfused with cold PBS through the right ventricle with cut vena cava until the pulmonary vasculature was cleared of blood. The whole lung was either excised for protein analyses or inflated with fixatives for histology. Airway mucus metaplasia was evaluated by Alcian blue staining and quantify as previously described [58].

### Histology evaluation and measurement of epidermal thickness and inflammation

Hematoxylin and eosin (H&E) were performed on skin sections after fixation with Streck solution (Streck Laboratories, St. La Vista, NE), as previously described [55]. Skin inflammatory cells and numbers of eosinophils and neutrophils were quantified by counting 8–10 fields (HPV at magnification ×200) per mouse (5–7 mice per group). Evaluation of epidermal thickness was performed as previously described [55]. H&E was performed on lung sections. The same microscopic magnification was used for sample slides from WT and IL-17A KO mice EC-sensitized with PBS, Ova, SEB, or Ova + SEB under comparison.

### Immunofluorescence analysis of Langerhans cells in the skin

To detect Langerhans cells, the skin section was analyzed as described previously [59]. Briefly, deparaffinized slides were blocked with donkey blocking solution for 1 hr. After washing, the slides were incubated with a goat anti-mouse Langerin antibody (Santa Cruz Biotechnology) at 4°C overnight. The slides were washed and incubated with an Alexa Fluor® 594 Donkey anti-goat IgG (Invitrogen) and DAPI (Roche Diagnostics) at room temperature for 2 hrs. After washing, the slides were mounted using PermaFluor (Thermo Scientific). The slides were evaluated using micrographs taken by a fluorescent microscope (Olympus BX-50) equipped with a camera (QImaging Retiga Exi). Langerin-positive cells were counted under the high power view and the data were the average of Langerhans cells in the skin of 5 different animals for each group.

### Stimulation and immune responses of spleen and draining lymph node (DLN) cells

Cell suspensions from spleen of each mouse, and pooled skin draining LN and bronchial LN cells of 3 mice for each group were prepared in complete RPMI-1640 (Invitrogen, Carlsbad, CA) supplemented with 10% FBS, 100 U/mL penicillin, and 100 μg/mL streptomycin. Cells were cultured at 4×10^6^/mL in 24-well plates in the presence of Ova 50 μg/mL or SEB 100 ng/mL or in anti-CD3 coated 96-well plates (BD Bioscience) with anti-CD28 (5 µg/ml). Supernatants were collected after 72 hrs of culture, and cytokines IL-4, IL-6, IFN-γ, IL-17A, and TGF-β were determined by ELISA following the manufacturer's instructions (R&D Systems, Minneapolis, MN).

### Statistical analysis of the data

Data are expressed as Mean ± SEM unless otherwise indicated. Data were analyzed using the Student's *t* test for comparison between two groups or ANOVA for comparison among multiple groups as appropriate. Difference with a value of P<0.05 was considered statistically significant.

## Supporting Information

Figure S1 **SEB enhanced Ova induced atopic dermatitis and atopic march.** (a) Epicutaneous sensitization and airway challenge protocol. Mice were sensitized with PBS, Ova (100 μg) and/or SEB (10 μg) on a sterile patch. Each mouse received a total of 3 one-week exposures to the patch, separated by two-week intervals. Airway Ova challenges and measurements were performed at the end of the third sensitization. (b) H&E staining of skin sections examined at magnification ×20. (c) Epidermal thickness (μm). (d) and (e) Skin inflammatory cells (HPF) and numbers of eosinophils and neutrophils by H&E per high power field at magnification ×40, respectively. Skin cytokine profile by ELISA: (f) IL-4, (g) IL-13 and (h) IFN-γ (n = 7 for each group; *p<0.05 and **p<0.01).
(TIF)

Figure S2 **SEB and Ova stimulated TGF-β1 and IL-23 production by lymphocytes and splenocytes of wild type mice after EC-sensitization.** The cells were stimulated with 50 (ng/ml) SEB or Ova (100 ng/ml) for 72 hrs and the supernatants were collected and used for measurement of TGF-β1 and IL-23 by ELISA. Levels of TGF-β1 produced by SEB-stimulated lymphocytes of DLNs (a) and spleen (b) and by Ova-stimulated lymphocytes of DLNs (e) and spleen (f). Levels of IL-23 produced by SEB-stimulated lymphocytes of DLNs (c) and spleen (d) and by Ova-stimulated lymphocytes of DLNs and spleen (g) and (h) (n = 5-6 mice per group).
(TIF)

Figure S3 **Levels of IL-6 in the serum and BAL.** (a) Serum levels of IL-6 and (b) Levels of IL-6 in the BAL fluids from PBS, SEB, Ova or Ova + SEB epicutaneously sensitized and challenged wild type mice. (n = 6–7 for each group; **p<0.01 and ***p<0.001).
(TIF)
