# Supplementary figures and images for: Epicutaneous Exposure to Staphylococcal Superantigen Enterotoxin B Enhances Allergic Lung Inflammation via an IL-17A Dependent Mechanism
Source: PLoS One. 2012 Jul 27;7(7):e39032. doi: 10.1371/journal.pone.0039032 (PMC3407176; doi:10.1371/journal.pone.0039032)

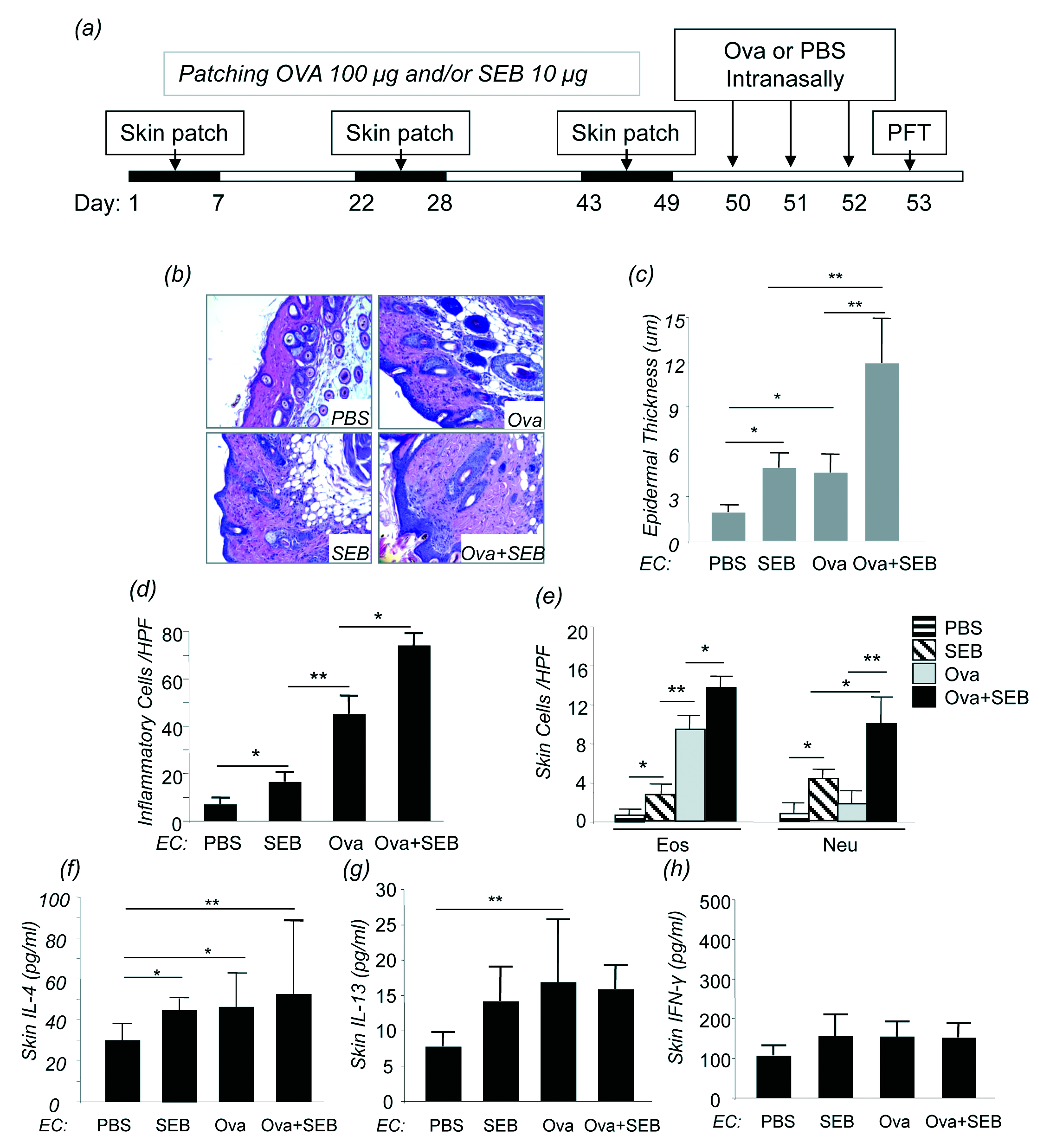

Supplement: Figure S1 — SEB enhanced Ova induced atopic dermatitis and atopic march. (a) Epicutaneous sensitization and airway challenge protocol. Mice were sensitized with PBS, Ova (100 μg) and/or SEB (10 μg) on a sterile patch. Each mouse received a total of 3 one-week exposures to the patch, separated by two-week intervals. Airway Ova challenges and measurements were performed at the end of the third sensitization. (b) H&E staining of skin sections examined at magnification ×20. (c) Epidermal thickness (μm). (d) and (e) Skin inflammatory cells (HPF) and numbers of eosinophils and neutrophils by H&E per high power field at magnification ×40, respectively. Skin cytokine profile by ELISA: (f) IL-4, (g) IL-13 and (h) IFN-γ (n = 7 for each group; *p<0.05 and **p<0.01). (TIF) [file pone.0039032.s001.tif]

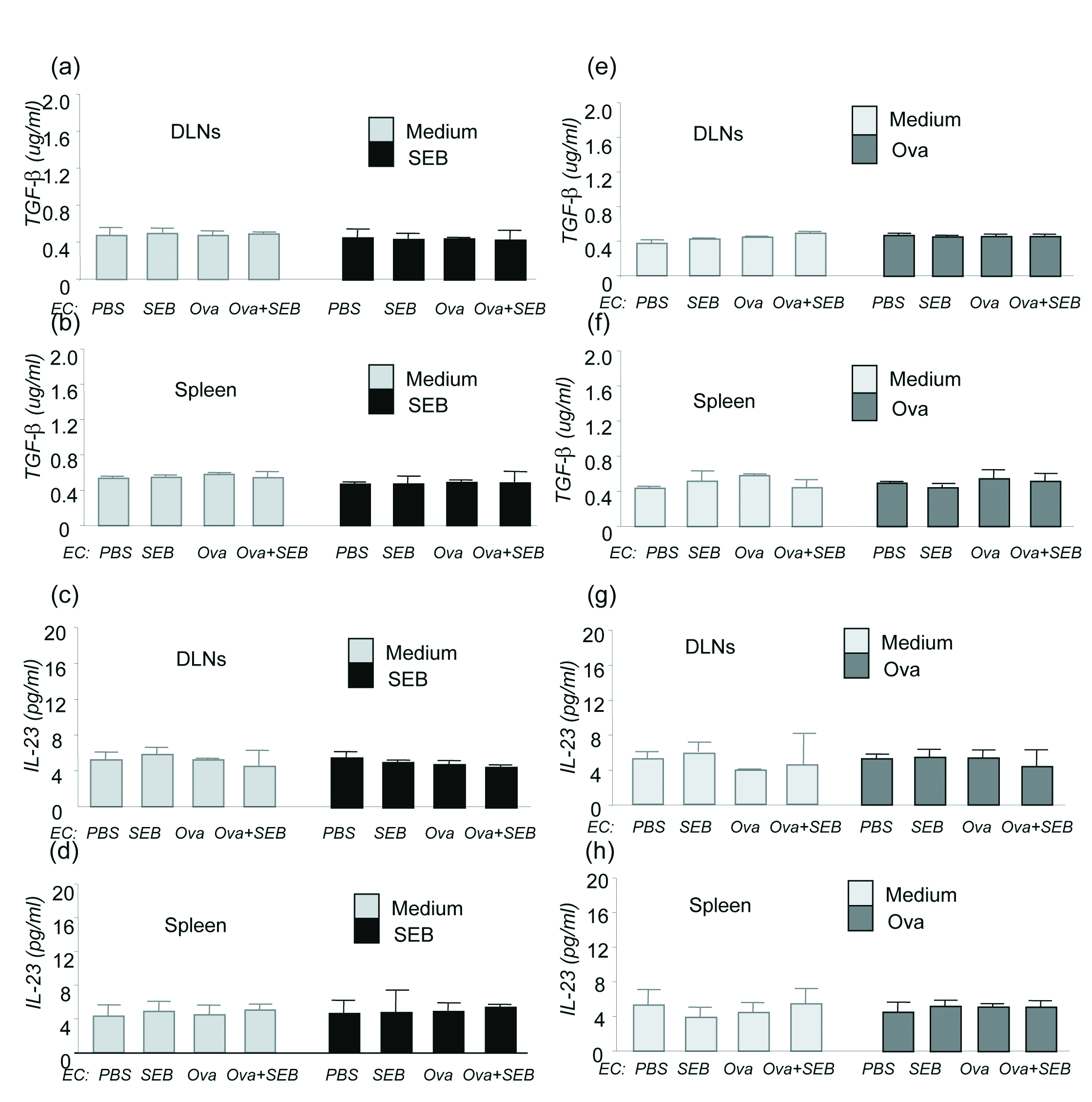

Supplement: Figure S2 — SEB and Ova stimulated TGF-β1 and IL-23 production by lymphocytes and splenocytes of wild type mice after EC-sensitization. The cells were stimulated with 50 (ng/ml) SEB or Ova (100 ng/ml) for 72 hrs and the supernatants were collected and used for measurement of TGF-β1 and IL-23 by ELISA. Levels of TGF-β1 produced by SEB-stimulated lymphocytes of DLNs (a) and spleen (b) and by Ova-stimulated lymphocytes of DLNs (e) and spleen (f). Levels of IL-23 produced by SEB-stimulated lymphocytes of DLNs (c) and spleen (d) and by Ova-stimulated lymphocytes of DLNs and spleen (g) and (h) (n = 5-6 mice per group). (TIF) [file pone.0039032.s002.tif]

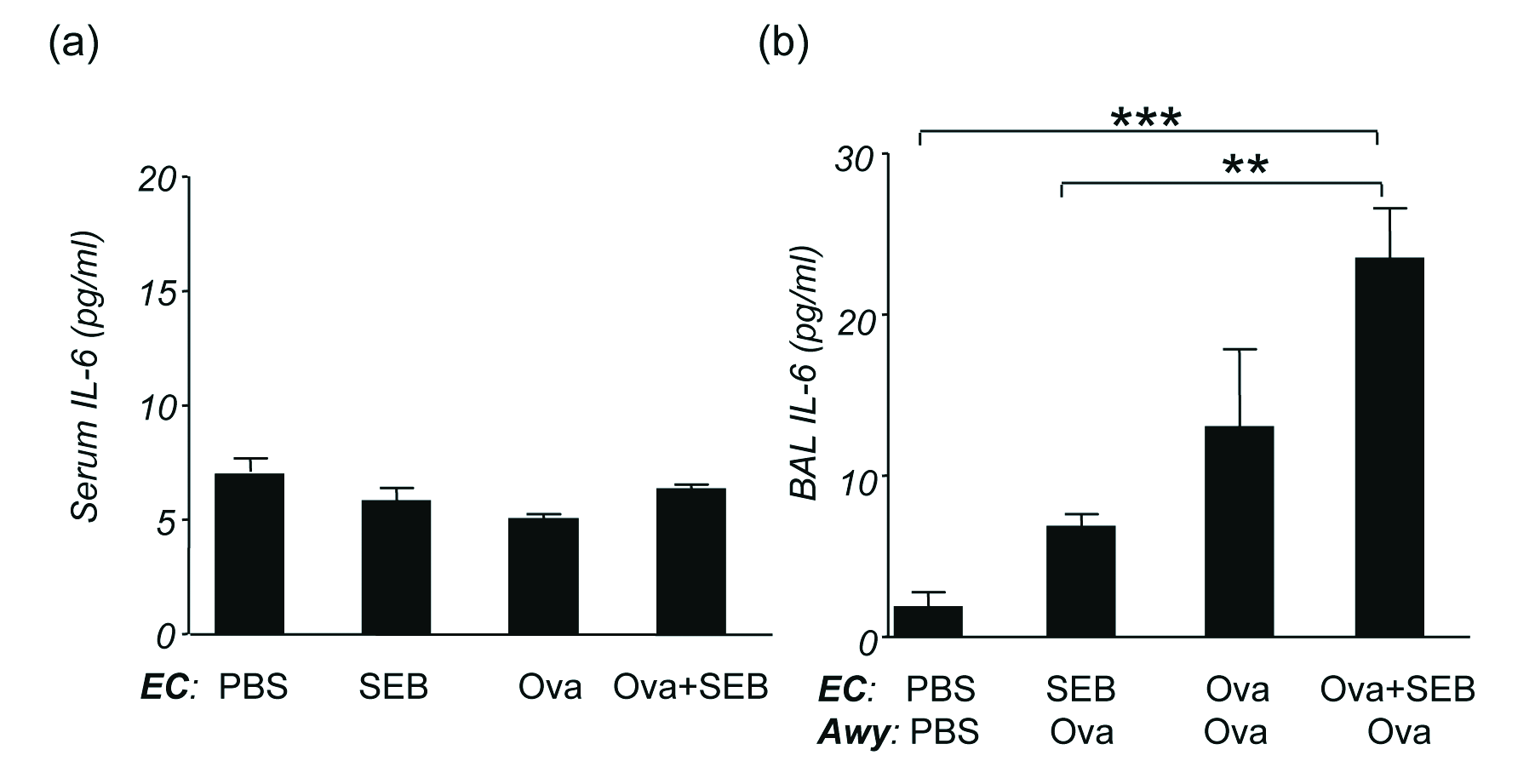

Supplement: Figure S3 — Levels of IL-6 in the serum and BAL. (a) Serum levels of IL-6 and (b) Levels of IL-6 in the BAL fluids from PBS, SEB, Ova or Ova + SEB epicutaneously sensitized and challenged wild type mice. (n = 6–7 for each group; **p<0.01 and ***p<0.001). (TIF) [file pone.0039032.s003.tif]
